# Insights Into the Mechanism of Tyrosine Nitration in Preventing β-Amyloid Aggregation in Alzheimer’s Disease

**DOI:** 10.3389/fnmol.2021.619836

**Published:** 2021-02-15

**Authors:** Jie Zhao, Qihui Shi, Ye Zheng, Qiulian Liu, Zhijun He, Zhonghong Gao, Qiong Liu

**Affiliations:** ^1^Shenzhen Key Laboratory of Marine Bioresource and Eco-environmental Sciences, College of Life Sciences and Oceanography, Shenzhen University, Shenzhen, China; ^2^Key Laboratory of Optoelectronic Devices and Systems of Ministry of Education and Guangdong Province, College of Optoelectronic Engineering, Shenzhen University, Shenzhen, China; ^3^Hubei Key Laboratory of Bioinorganic Chemistry and Materia Medica, School of Chemistry and Chemical Engineering, Huazhong University of Science and Technology, Wuhan, China; ^4^Shenzhen Bay Laboratory, Shenzhen, China

**Keywords:** tyrosine nitration, Aβ, Alzheimer’s disease, fibrilization, protein modification

## Abstract

Nitration of tyrosine at the tenth residue (Tyr10) in amyloid-β (Aβ) has been reported to reduce its aggregation and neurotoxicity in our previous studies. However, the exact mechanism remains unclear. Here, we used Aβ_1–42_ peptide with differently modified forms at Tyr10 to investigate the molecular mechanism to fill this gap. By using immunofluorescent assay, we confirmed that nitrated Aβ was found in the cortex of 10-month-old female triple transgenic mice of Alzheimer’s disease (AD). And then, we used the surface-enhanced Raman scattering (SERS) method and circular dichroism (CD) to demonstrate that the modification and mutation of Tyr10 in Aβ have little impact on conformational changes. Then, with the aids of fluorescence assays of thioflavin T and 4,4′-dianilino-1,1′-binaphthyl-5,5′-disulfonic acid, transmission electron microscopy (TEM), atomic force microscopy (AFM), and dynamic light scattering (DLS), we found that adding a large group to the phenolic ring of Tyr10 of Aβ could not inhibit Aβ fibrilization and aggregation. Nitration of Aβ reduces its aggregation mainly because it could induce the deprotonation of the phenolic hydroxyl group of Tyr10 of Aβ at physiological *pH*. We proposed that the negatively charged Tyr10 caused by nitration at physiological *pH* could interact with the salt bridge between Glu11 and His6 or His13 and block the kink around Tyr10, thereby preventing Aβ fibrilization and aggregation. These findings provide us new insights into the relationship between Tyr10 nitration and Aβ aggregation, which would help to further understand that keeping the balance of nitric oxide *in vivo* is important for preventing AD.

## Introduction

Alzheimer’s disease (AD) is a neurodegenerative disorder leading to severe memory deficits, progressive cognitive decline, and neuronal death (Miranda et al., 2000; Selkoe and Podlisny, 2002; Goedert and Spillantini, 2006). Its pathophysiology involves extracellular amyloid plaque, in which the primary protein constituents aggregated Aβ (Querfurth and Laferla, 2010). Aβ is derived from amyloid precursor protein (APP) through sequential proteolytic cleavages by β- and γ-secretases, with Aβ_1–40_ and Aβ_1–42_ as the predominant species (Roher et al., 1993; Lambert et al., 1998; LaFerla et al., 2007). Under pathological conditions, accumulated Aβ peptides can aggregate into oligomers, protofibrils, and mature fibrils after conformational changes from α-helix to β-sheet (Karran et al., 2011; Knowles et al., 2014). Moreover, the soluble species of oligomers and protofibrils have been proposed as the primary driving force for AD formation in the amyloid hypothesis (Klein, 2002; Selkoe, 2008; Henry et al., 2015). Excitingly, aducanumab, a drug that selectively targets aggregated Aβ and reduces brain Aβ in a dose-dependent manner, has been proven effective in reducing the clinical decline in patients with early AD after analysis of a large data set (Sevigny et al., 2016). Therefore, a detailed understanding of the pathological process of Aβ self-assembly on a molecular level is of fundamental importance to elucidate the risk factors associated with the progression of AD and to develop new and effective intervention strategies.

It has been reported that multiple posttranslationally modified (PTM) Aβ peptides were found in brains of AD patients (Jawhar et al., 2011; Kummer and Heneka, 2014), including the E3- and E11-pyroglutarmation (Perez-Garmendia et al., 2010; Rijal Upadhaya et al., 2014), the D7-isomerization (Moro et al., 2018), the S8-phosphorylation (Kumar et al., 2013; Rijal Upadhaya et al., 2014), the S26-phosphorylation (Kumar et al., 2016), and the Y10-nitration of Aβ (Kummer et al., 2011). The isoaspartate and pyroglutamate-modified amyloid-β are known to show increased hydrophobicity, higher toxicity, faster aggregation, and β-sheet stabilization and are more resistant to degradation. It plays an important role in triggering neurodegeneration and lethal neurological deficits (Wirths et al., 2009; Moro et al., 2018). Phosphorylation of serine residue eight in Aβ promotes the formation of oligomeric Aβ assemblies and increases its toxicity in Drosophila models (Kumar et al., 2013). Phosphorylation of Aβ at Ser26 is also found to strongly promote the formation and stabilization of Aβ oligomers with increased toxicity on human neurons (Kumar et al., 2016). Phosphorylation of Aβ is believed to be closely associated with the pathogenesis of the most common sporadic form of AD. Therefore, elucidating the effects of post-translational modifications on the molecular properties of Aβ aggregates may contribute to understanding the relationship between Aβ misfolding and AD.

Tyrosine nitration is a frequent post-translational modification that would increase the amino acid size by incorporation of a large substituent and cause a remarkable decrease in the ionization constant (*pK*_a_) of the phenolic hydroxyl group from 10.1 to the value around 7 in aqueous solution, leading to deprotonation of the phenolic hydroxyl group at physiological *pH* (Zamyatnin, 1972; De Filippis et al., 2006; Abello et al., 2009). It has been reported that protein tyrosine nitration could significantly affect the function of some essential proteins, and accumulated evidence shown that many critical functional proteins are significantly nitrated in AD (Giasson et al., 2000; Sultana et al., 2006; Pacher et al., 2007; Alkam et al., 2008). Interestingly, there is a tyrosine residue at position 10 of Aβ, which is reported to be active toward copper and heme-binding and plays crucial roles in nitration and phosphorylation of Aβ (Wise and Coskuner, 2014; Lu et al., 2015; Yeo et al., 2015; Perluigi et al., 2016). Furthermore, Kummer et al. (2011) reported that nitrated Aβ was detected in the core of plaques of APP/PS1 double transgenic mice and brains of AD patients, and Guivernau et al. (2016) also observed nitrotyrosine immunoreactivity in the plaques of AD patients’ brain, indicating that Aβ can be nitrated *in vivo*. Our recent studies found that nitration of Aβ can significantly inhibit its fibril formation and reduce its toxicity toward neurons (Zhao et al., 2015, 2017).

Moreover, nitration of Aβ can significantly inhibit its toxic oligomer formation induced by copper ions. We proposed that nitration of Aβ may be an essential protective mechanism for its normal function (Zhao et al., 2019). While it is still unclear how nitration of Aβ inhibits its fibril formation because tyrosine nitration can change the amino acid size and the pKa value of the phenolic hydroxyl group. Exploring the mechanisms underlying Aβ aggregation and searching for the modulating factors is critical to understand the role of Aβ in the development of AD.

In this study, we designed various Tyr10-modified or mutated Aβ_1–42_ to systematically investigate the mechanism for nitration of Aβ in inhibiting its primary nucleation and subsequent fibrilization and aggregation. To figure out the underlying mechanism, we designed the following Tyr10 modified or mutated Aβ peptides: Aβ_1–42_NT (in which Tyr10 is replaced with 3-nitro-tyrosine), Aβ_1–42_DM (in which Tyr10 is replaced with 2,6-dimethyl-tyrosine), Aβ_1–42_I (in which Tyr10 is replaced with 3-iodo-tyrosine), Aβ_1–42_Cl (in which Tyr10 is replaced with 3-chloro-tyrosine) and Aβ_1–42_NF (in which Tyr10 is replaced with 3-nitro-phenylalanine). We used Aβ_1–42_DM to test the effect of steric hindrance on the aggregation of Aβ_1–42_ because 2,6-dimethyl-tyrosine is bigger than tyrosine and the *pKa* value of the phenolic hydroxyl group in 2,6-dimethyl-tyrosine is higher than that of tyrosine. Aβ_1–42_Cl and Aβ_1–42_I were used to investigate the effect of *pK_a_* change because of their different *pK_a_* values of the phenolic hydroxyl group. The Aβ_1–42_NF was used to test the hydrogen-bond interaction of the phenolic hydroxyl group in Tyr10 of Aβ_1–42_NT. The influence of Tyr10 modification and mutation on Aβ nucleation and fibrilization, fibril morphology, and secondary structure are assessed by surface-enhanced Raman scattering (SERS), circular dichroism (CD), fluorescence assays of thioflavin T (ThT) and 4,4′-dianilino-1,1′-binaphthyl-5,5′-disulfonic acid (Bis-ANS), transmission electron microscopy (TEM), atomic force microscopy (AFM) and dynamic light scattering (DLS). These results suggest that the *pK_a_* changes of the phenolic hydroxyl group in Tyr10 induced by nitration of Aβ play a crucial role in its inhibitory effect. The effect of Aβ nitration on its structure and function deserves more attention in anti-AD drug development.

## Materials and Methods

### Materials

Aβ_1–42_, Aβ_1–42_NT, Aβ_1–42_DM, Aβ_1–42_I, Aβ_1–42_Cl, and Aβ_1–42_NF (>95%, lyophilized powder) are synthesized by China Peptides (Shanghai, China). Thioflavin T (ThT), 4,4′-dianilino-1,1′-binaphthyl-5,5′-disulfonic acid (Bis-ANS) are obtained from Sigma–Aldrich (St. Louis, MO, USA). All other reagents are in the analytical grade. The female triple transgenic model mice of AD carrying human gene mutants APPswe, PS1M146V, and tauP301L are purchased from the Jackson Laboratory (Bar Harbor, ME, USA).

### Immunofluorescence Analysis

The 10 μm PFA-fixed and mounted brain sections of female triple transgenic model mice of AD were obtained and prepared using the methods described previously (Xie et al., 2018). The experiments and procedures were performed following the institutional guidelines regarding experimental animal use at Shenzhen University and were approved by the Animal Ethical and Welfare Committee of Shenzhen University (permit number AEWC-20140615-002). Briefly, the brain sections were dewaxed and rehydrated through a series of xylene and ethanol incubations and then underwent boiling antigen retrieval in citrate buffer (10 mM sodium citrate, *pH* 6) and finally washed with PBS three times for 5 min. For immunofluorescent experiments, sections were blocked in 5% bovine serum albumin in PBS with 0.2% Triton for 30 min, after which they were immunofluorescently stained with 1:500 polyclonal rabbit anti-nitrotyrosine antibody (3NT; Cell Signaling Technology) and 1:200 6E10 mouse monoclonal anti-Aβ antibody in blocking solution overnight at 4°C followed by 1:1,000 Alexa-488-bound anti-rabbit and 1:1,000 Alexa-555-bound anti-mouse as secondary antibodies at room temperature (RT) for 1 h. Afterward, sections were washed and incubated with DAPI (Sigma) for 20 min to visualize nuclei. Finally, sections were washed and coverslipped using PermaFluor^TM^ Aqueous Mounting Medium (Thermo Fisher Scientific, Waltham, MA, USA). Samples were visualized using a Leica SPE fluorescent microscope.

### Aβ Preparation

To remove pre-existing aggregates, Aβ and mutants were dissolved in 1,1,1,3,3,3-hexafluoroisopropanol (HFIP) overnight, and then the solution was centrifugated at 12,000 rpm for 15 min to remove the aggregates. A lyophilizer then removed the solvent, and the peptide was stored as a solid at −20°C until used. Also, the lyophilized Aβ peptides were dissolved to 5 mM in 10 mM NaOH solution and further diluted to the desired concentration with 20 mM phosphate buffer solution (PB), *pH* 7.4 before use [the concentrations of Aβ_1–42_ and its mutants in PB were determined using Bicinchoninic acid (BCA) protein assay].

### Surface-Enhanced Raman Spectroscopy

The SERS analysis was performed on a Renishaw System 1000 Raman Spectrometer (Wotton-under-Edge, UK) with an excitation wavelength at 532 nm. A 50× (NA = 0.75) air objective lens was used to deliver 8 mW to the sample with an approximate 2.2 μm spot size. For SERS measurements, 100 μM Aβ or mutants was incubated in 5 mM PB (pH 7.4) at 37°C for 48 h and then mixed with AgNPs in equal proportions. The mixture (5 μl) was deposited onto a silicon slice and dried at RT. Raman scans were integrated for 30 s over the range of 1,200–1,300 cm^−1^.

### Circular Dichroism Spectroscopy

The CD spectra of Aβ (20 μM) and mutants (20 μm) in 5 mM PB (pH 7.4) at 0 and 48 h were recorded from 190 to 260 nm on a JASCO-810 (Tokyo, Japan) spectropolarimeter at RT using a quartz cuvette with a path length of 1 mm. The bandwidth was 1 nm and the scan speed was set at 100 nm/min. The background signal from the PB buffer has been subtracted by running PB alone as a blank and the data were averaged after being measured three times.

### ThT and Bis-ANS Fluorescence Assay

ThT fluorescence assay was performed to detect the fibril formation of Aβ and mutants, as an increase in fluorescence emission at 485 nm occurs upon the amyloid-specific dye ThT incorporation into β-sheet amyloid structures (LeVine, 1999). The hydrophobic probe Bis-ANS, which can bind to hydrophobic patches formed due to aggregation and induce an increase in fluorescence emission at 496 nm (Rosen and Weber, 1969), was also used to determine the accumulation of Aβ and mutants. All the fluorescence assay was performed on a Synergy H1 multi-mode microplate reader (Bio-Tek Instruments Inc., Winooski, VT, USA) with a Nunc 96-well flat-bottom, black polystyrene microplate (Thermo Fisher Scientific). For the measurement, Samples (50 μM) were incubated with 50 μM ThT or Bis-ANS in PB at 37°C for 48 h. The ThT fluorescence was measured every 60 min using an excitation wavelength at 440 nm. The Bis-ANS fluorescence was tested at 48 h using an excitation wavelength at 385 nm.

### Transmission Electron Microscopy

The samples for TEM observation were prepared by incubating Aβ (50 μM) and mutants (50 μM) in PB (pH 7.4) at 37°C for 48 h. Then, 20 μl of each sample was dropped on a 200-mesh copper grid covered by carbon-stabilized Formvar film and allowed to absorb 10 min. The excess sample solution was removed, and grids were then washed three times with deionized water. Finally, the grids were stained with 5% uranyl acetate for 5 min and air-dried. Images were obtained using a transmission electron microscope (HITACHI H-7000FA) at an accelerating 30 kV voltage.

### Atomic Force Microscopy

The samples for AFM observation were prepared, as indicated above. A 30 μl solution of each sample (50 μM) was deposited on freshly cleaved, highly oriented pyrolytic graphite. After an absorption time of 10 min, the excess solution was removed, and the highly oriented pyrolytic graphite was washed with deionized water, dried at RT. AFM measurements were taken on a Multimode 8 AFM (Bruker, Billerica, MA, USA) with ScanAsyst-Fluid cantilevers (Bruker, Billerica, MA, USA) in ScanAsyst mode. At least four mica surface regions were examined to ensure that similar structures existed throughout the sample.

### Dynamic Light Scattering

A laser diffraction particle size analyzer (Beckman Coulter LS230, USA) was used to determine the particle size distribution of aggregates at 20°C using a small volume (40 μl) quartz cuvette of 1 cm path. A total of 100 μm Aβ and mutants were incubated in 10 mM PB pH 7.4 at 37°C for 24 h. Scattering data were collected as an average of three measurements with six scans for each measurement.

### Statistical Analysis

All of the experiments were repeated at least three times. The results were expressed as the means ± SE.

## Results

### Modification and Mutation of Aβ Peptides

**Figure 1:**
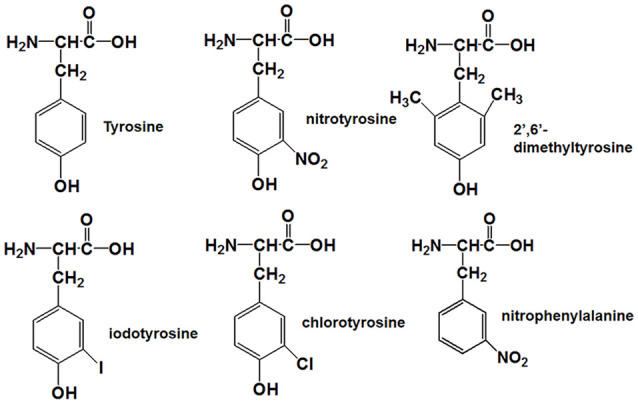
Molecular structures of tyrosine, nitrotyrosine, 2′,6′-dimethyltyrosine, iodotyrosine, chlorotyrosine, and nitrophenylalanine.

Tyrosine nitration alters the bulkiness of the residue, which becomes 30 Å larger than the 205 Å of nonmodified tyrosine, and also induces a significant decrease of the *pK_a_* in the phenolic hydroxyl group from 10.1 to the value around 7 in aqueous solution, resulting in the fact that the hydroxyl group of nitrotyrosine is about 50% charged at physiological *pH* (Zamyatnin, 1972; De Filippis et al., 2006). Also, the nitro group may have effects on the structure of Aβ through hydrogen bond interactions. Our previous research proposed that nitration of Aβ might inhibit its aggregation by interrupting its intermolecular interactions, and it still needs to be confirmed (Zhao et al., 2017). To figure out the underlying mechanism, the following Tyr10 modified and mutated Aβ peptides were designed and synthesized: Aβ_1–42_DM for testing the effect of steric hindrance, Aβ_1–42_Cl and Aβ_1–42_I for investigating the effect of *pK_a_* change (due to their different *pK_a_* values of phenolic hydroxyl group), Aβ_1–42_NF as a control for testing the effect of the phenolic hydroxyl group in Tyr10 of Aβ_1–42_NT and its hydrogen bond formed with the nitro group. The molecular structures of the amino acid in the tenth position of Aβ_1–42_ were shown in Figure 1. Table 1 shows the physicochemical property (Van der Waals volume (VW), distribution coefficient (LogD), and ionization constant (*pK_a_*) of the amino acid in the tenth position of Aβ_1–42_ calculated by the computer program ChemAxon, based on atom/fragment contributions. The percent deprotonation of the phenolic hydroxyl group of tyrosine/modified tyrosine in phosphate buffer (PB, pH 7.4) was calculated by the Henderson-Hasselbalch (HH) equation (Avdeef, 2007; Sugano et al., 2007).

**Table 1:** The physicochemical property and ionization constant of the amino acid in the tenth position of Aβ_1–42_ peptide.

Aβ variants	VW (Å^3^)	Log D	*pKa*	Percent deprotonation (%)
Aβ_1–42_	164.03	−1.5	9.79	0.40
Aβ_1–42_NT	187.06	−2.32	6.69	84
Aβ_1–42_DM	198.13	−0.47	9.94	0.29
Aβ_1–42_I	188.59	−0.61	8.34	10
Aβ_1–42_Cl	177.98	−1.0	7.92	23
Aβ_1–42_NF	178.61	−1.25	-	-

### Detection of Nitrated Aβ in the Brain of Triple Transgenic AD Mouse

**Figure 2:**
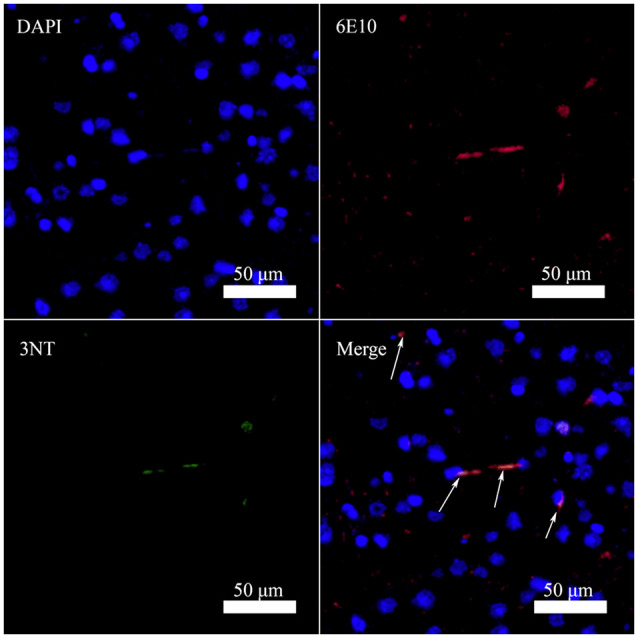
Nitrated Aβ is detectable in the cortex of 10-month-old female triple transgenic model mice of Alzheimer’s disease (AD) using Aβ antibody 6E10 (Aβ) and 3-nitrotyrosine antibody (3NT; bar = 50 μm).

It has been reported that Aβ can bind to heme and copper ions and increase the peroxidase activity of the complex (Lu et al., 2015). Consequently, the Tyr10 in Aβ is prone to nitration in the presence of nitrite and hydrogen peroxide *in vitro* (Yuan and Gao, 2013; Lu et al., 2015). To determine whether Aβ can be nitrated *in vivo*, we performed Immunofluorescence analysis of nitrotyrosine and Aβ in brain sections of a 10-month-old female triple transgenic model mouse of AD. Analysis of brain sections revealed colocalization of antibody 6E10 against Aβ with the nitrotyrosine antibody 3NT in this AD model mouse (Figure 2), indicating that Aβ could be nitrated *in vivo*. These results were in good agreement with the observation reported by Kummer et al. (2011) and Guivernau et al. (2016), and also suggested an important role for nitration of Aβ in AD. Those spots stained by 6E10 but not by 3NT in Figure 2 are small Aβ plaques without Tyr10 nitration. We suggest that nitrated Aβ can only be trapped in large plaques.

### Secondary Structure Analysis of Aβ and Its Mutants by SERS and CD

**Figure 3:**
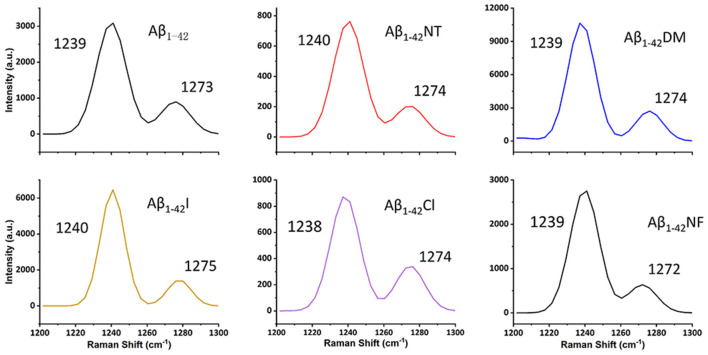
Surface-enhanced raman scattering (SERS) spectra of Aβ_1–42_ (100 μM) and mutants (100 μM) aggregates. After 48 h incubation in phosphate buffer (PB) at 37°C, the samples were mixed with nanostructured silver and dropped on a silicon slide, and dried at room temperature (RT). Raman scans were integrated for 30 s over the range of 1,200–1,300 cm^−1^ with excitation at 532 nm. The spectra were averaged over three separate measurements.

It is commonly accepted that conformational changes of Aβ from α-helix to β-sheet is critical to its aggregation. In previous studies, we confirmed that both nitration and chlorination of Aβ have little effect on its β-sheet formation and proposed that nitration of Aβ inhibits its aggregation by disrupting its intermolecular interactions (Zhao et al., 2017). Herein, SERS was used to detect the effect of Tyr10 modification and mutation of Aβ on its secondary structure. SERS is a very useful and efficient tool for detecting conformational changes of Aβ by quantitatively analyzing its characteristic amide III region frequencies around 1,200–1,300 cm^−1^ (Mikhonin et al., 2006; Oladepo et al., 2011; Wang et al., 2013). According to the previous researches, the peaks at 1,279 and 1,241 cm^−1^ are related to α-helix and β-sheet secondary structure, respectively (Mikhonin et al., 2006). Moreover, the strength ratios for the peaks can be employed to determine the protein secondary structures (Tian et al., 2019). We measured the area of the peaks at 1,279 and 1,241 cm^−1^ to calculate the percentage of the secondary structure of the samples. The percentages of different secondary structures of the samples are listed in Table 2. As shown in Figure 3, all samples exhibited a strong peak at 1,241 cm^−1^ and a weak peak at 1,279 cm^−1^, indicating that Tyr10 modification and mutation of Aβ has little impact on its conformational changes.

**Table 2:** Percentages of different secondary structures of the sample, obtained by the analysis of the amide III region.

Aβ variants	α helix (%)	β sheet (%)
Aβ_1–42_	20.2	79.8
Aβ_1–42_NT	21.1	78.9
Aβ_1–42_DM	19.9	80.1
Aβ_1–42_I	22	78
Aβ_1–42_Cl	28	72
Aβ_1–42_NF	18.5	81.5

**Figure 4:**
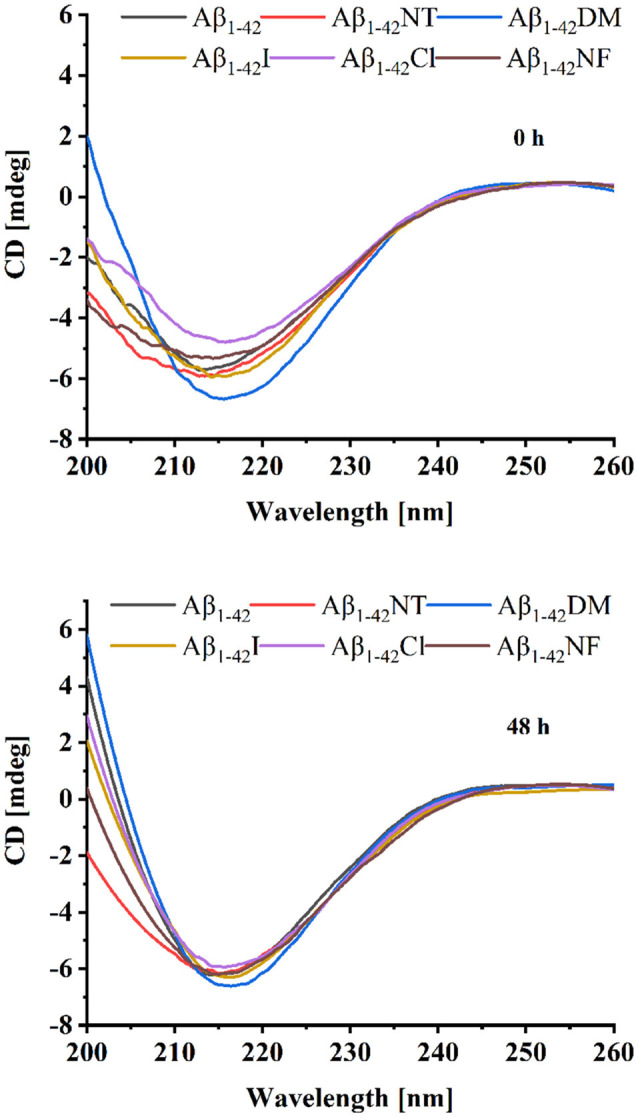
Circular dichroism (CD) spectra of Aβ (20 μM) and mutants (20 μM) at 0 and 48 h. The background signal from the PB buffer has been subtracted by running PB alone as a blank and the data were averaged after being measured three times.

We also used CD to detect the secondary structure of samples at 0 and 48 h. As shown in Figure 4, the CD spectrum of Aβ_1–42_ and mutants had a negative peak around 217 nm at 0 h, which is considered typical for a β-sheet structure. It indicated that Aβ_1–42_ and mutants had aggregated at 0 h. There is little difference between the four peptides (Aβ_1–42_, Aβ_1–42_NT, Aβ_1–42_I, and Aβ_1–42_NF). The Aβ_1–42_DM showed the largest negative peak at 217 nm. It indicated that methylated modification of tyrosine in Aβ could promote its conformational changes from α helix to β-sheet. The Aβ_1–42_Cl showed a minimal negative peak at 217 nm. This result was consistent with that obtained from SERS. It suggested that chlorination of Aβ at tyrosine could affect its conformational changes. After 48 h incubation, the negative CD values of samples increased. Similarly, the Aβ_1–42_DM showed the largest negative peak at 217 nm and the Aβ_1–42_Cl showed a minimal negative peak at 217 nm. Also, there is no significant difference between the samples. These results were consistent with those obtained from SERS.

### Effect of Tyr10 Modification and Mutation on the Aggregation of Aβ

**Figure 5:**
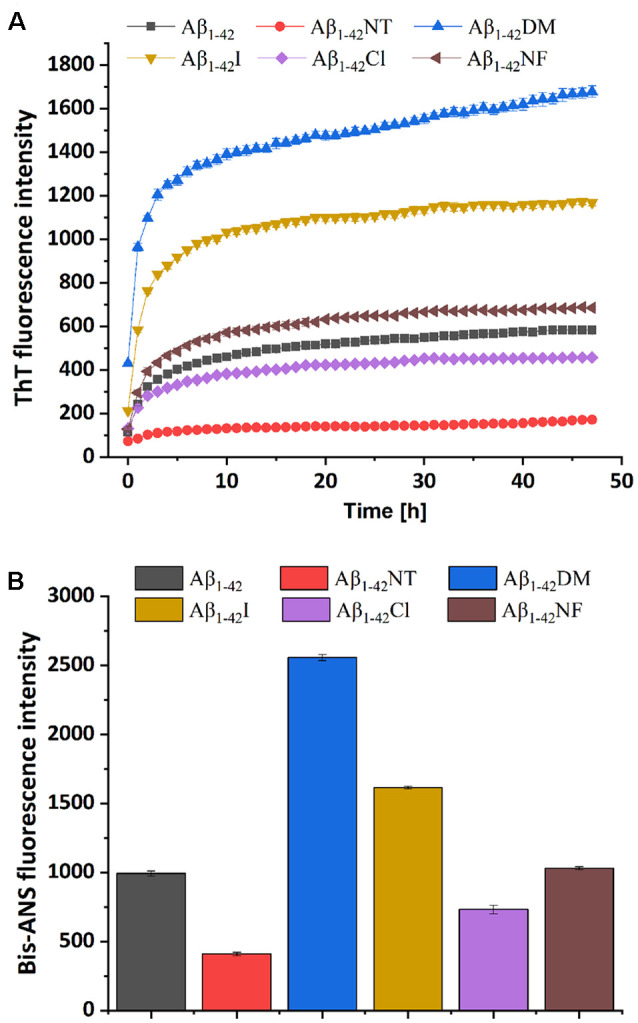
Aggregation of Aβ_1–42_ and mutants was monitored by ThT and Bis-ANS fluorescence assay. **(A)** The kinetics process of fibril formation for Aβ_1–42_ and its mutants in 50 mM PB (*pH* 7.4) at 37°C was detected by ThT fluorescence assay. **(B)** After 48 h incubation in 50 mM PB (*pH* 7.4) at 37°C, the aggregation degree of the samples was measured by Bis-ANS fluorescence assay. The values are subtracted by that of ThT or Bis-ANS control. For the measurement, the peptide concentration is 50 μM, and the concentration of ThT or Bis-ANS fluorescence dye was 50 μM and 25 μM, respectively. All the experiments were repeated at least three times, and values are the mean ± SE of triplicate experiments.

ThT fluorescence assay is useful to assess the content of β-sheet-rich structures quantitatively and is widely used to evaluate Aβ fibrilization and aggregation (LeVine, 1999). As shown in Figure 5A, Aβ_1–42_ showed a significant increase in fluorescence values that reached the plateau stage after about 10 h incubation, suggesting a rapid fibrillation process. In contrast, the fluorescent intensity of the Aβ_1–42_NT group was significantly lower than that of Aβ_1–42_, which indicated a lower degree of aggregation in Aβ_1–42_NT. These results are consistent with our previous results (Zhao et al., 2017). We did not find a lag phase in the aggregation curves of Aβ_1–42_NT, suggesting that nitration of Aβ_1–42_ has little effect on monomers’ nucleation. It is well known that hydrogen bond interactions are important for maintaining the structure of the protein. Thus, a question was raised about whether nitration of Aβ inhibits its aggregation through hydrogen bonds because the nitro group is easy to form hydrogen bonds with other amino acids. Therefore, we used Aβ_1–42_NF to test the possible effect of the hydrogen bond. Comparing the results of Aβ_1–42_NF and Aβ_1–42_, we found that there is no significant difference in fluorescent intensity between Aβ_1–42_NF and Aβ_1–42_, indicating that the hydrogen bond formed between the phenolic hydroxyl group and the nitro group inside the nitrotyrosine residue is not necessary for the inhibitory effect of nitrated Aβ. Moreover, the volume of Aβ_1–42_NF (178.61 Å^3^) is bigger than that of Aβ_1–42_ (164.03 Å^3^), indicating that increasing the volume of the substituent group in the phenolic ring of tyrosine does not affect the aggregation of Aβ. The percent deprotonation of nitrotyrosine, chlorotyrosine, and iodotyrosine is 84%, 23%, and 10%, respectively. According to the aggregation of the three peptides, the highest fluorescence intensity was observed for Aβ_1–42_I, followed by Aβ_1–42_Cl and Aβ_1–42_NT, suggesting that the *pK_a_* changes of the phenolic hydroxyl group may be critical for the inhibitory effect (the *pK_a_* values of the phenolic hydroxyl group of iodotyrosine, chlorotyrosine and nitrotyrosine are 8.34, 7.92, and 6.9, respectively), and further confirmed that increasing the volume of the substituent group in the phenolic ring of tyrosine has little effect on the aggregation of Aβ since that iodine has greater volume compared with chlorine (the VW values of iodotyrosine and chlorotyrosine are 188.59 and 177.98 Å^3^, respectively). It also indicated that tyrosine chlorination could inhibit the aggregation of Aβ.

It has been reported that bis-ANS is an excellent alternative to ThT for monitoring fiber formation kinetics *in vitro*. It is more sensitive to fiber detection than ThT (Younan and Viles, 2015). To further confirm the observations obtained from the ThT fluorescence assay, the Bis-ANS fluorescence assay was also applied to study the effect of Tyr10 modification and mutation on Aβ aggregation. Bis-ANS is a hydrophobic fluorescent probe widely used to study peptide folding. It can specifically bind to the solvent-exposed hydrophobic surface, leading to increased fluorescence emission and a blue shift of the emission maximum (Rosen and Weber, 1969). As can be seen from Figure 5B, Bis-ANS analysis showed similar results as that of ThT fluorescence assay that increasing the steric hindrance in the phenolic ring of Tyr10 did not affect Aβ aggregation and the change of *pK_a_* of the hydroxyl group in Tyr10 is critical for the inhibitory effect of nitration of Aβ on its aggregation.

### Morphological Study of Tyr10 Modification and Mutation on Aβ Aggregation

**Figure 6:**
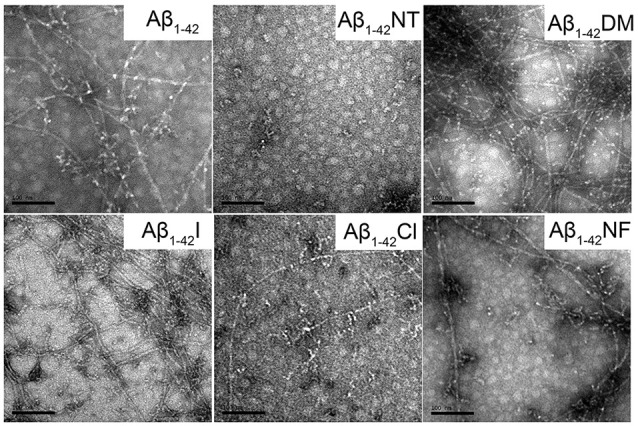
Negative-stained transmission electron microscopy (TEM) images of Aβ_1–42_ and mutants. After 48 h incubation in 50 mM PB (*pH* 7.4) at 37°C, the morphologies of samples were detected by TEM. The scale bar is 100 nm, and the peptide concentration is 50 μM.

To visually observe the effect of Tyr10 modification and mutation on the fibrillation of Aβ, TEM was used to evaluate the morphology of Aβ and mutants’ fibrils. As shown in Figure 6, after incubation in phosphate buffer (PB; *pH* 7.4) at 37°C for 48 h, Aβ_1–42_, Aβ_1–42_DM, Aβ_1–42_I, and Aβ_1–42_NF aggregated into numerous massive and crowded mature amyloid fibrils, especially the Aβ_1–42_DM and Aβ_1–42_I. However, few fibrils were observed in the images of Aβ_1–42_Cl, suggesting that Aβ_1–42_Cl has lower aggregation propensities. There are no fibrils in the TEM image of Aβ_1–42_NT, indicating that Aβ_1–42_NT could not form fibrils. This result was consistent with our previous studies (Zhao et al., 2017).

**Figure 7:**
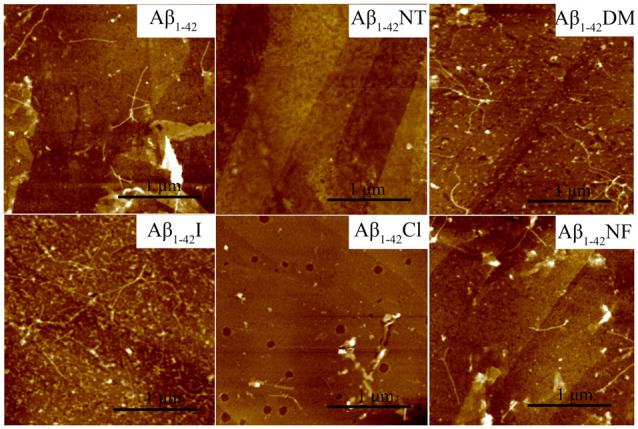
Atomic force microscopy (AFM) images of Aβ_1–42_ and mutants. After 48 h incubation in 50 mM PB (*pH* 7.4) at 37°C, the morphology of samples was detected by AFM. The scale is 1 μm, and the peptide concentration is 50 μM.

The influence of Tyr10 modification and mutation on Aβ fibrils’ morphology was further confirmed by AFM widely used to observe amyloid peptides’ morphology. As expected, a lot of mature amyloid fibrils were observed in the images of Aβ_1–42_, Aβ_1–42_DM, Aβ_1–42_I, and Aβ_1–42_NF, especially the Aβ_1–42_DM and Aβ_1–42_I, and few fibrils were found in the images of Aβ_1–42_Cl (Figure 7). Similarly, no fibrils were observed in the AFM images of Aβ_1–42_NT. The TEM and AFM data agree with that obtained from ThT and Bis-ANS fluorescence assay, further confirming that *pKa* changes of the phenolic hydroxyl group in Tyr10 induced by tyrosine nitration may play an important role in its inhibitory effect on Aβ fibrils formation.

### Characterization of the Size Distributions of Aβ_1–42_ and Its Mutants by Dynamic Light Scattering (DLS)

**Figure 8:**
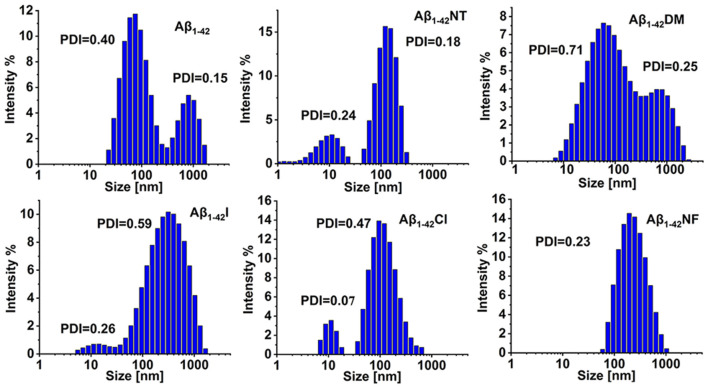
Analysis of the aggregates size distributions of Aβ_1–42_ and mutants by dynamic light scattering (DLS). The DLS samples were prepared by incubating Aβ_1–42_ (100 μM) and mutants (100 μM) in 5 mM PB (*pH 7.4*) at 37°C for 24 h. The values of the polymer dispersity index (PDI) of the samples are less than 1, indicating these samples are uniform and suitable for DLS. All the experiments were repeated at least three times.

To further confirm the results obtained above, DLS was used to monitor the size distributions of Aβ_1–42_ and mutants aggregates. From Figure 8, we can see that all the samples are very polydisperse after 24 h incubation in 5 mM PB (*pH 7.4*) at 37°C, indicative of the existence of several size species. The particle size distribution of Aβ_1–42_ comprised two peaks, one at 88 nm and the other centered at 861 nm. In contrast, the size of Aβ_1–42_NT was found to be around 136 nm, much less than the size distribution peak of Aβ_1–42_, indicating that nitration of Aβ can significantly inhibit its aggregation. For the Aβ_1–42_Cl, the dominant species is centered at 140 nm, and more large species were observed compared with Aβ_1–42_NT. For Aβ_1–42_NF, a peak of size distribution showed up around 233 nm, indicating that the hydrogen bond formed between the phenolic hydroxyl group and the nitro group is unnecessary for the inhibitory effect of Tyr10 nitration on Aβ aggregation. It also should be noted that there is a single population in the DLS spectrum of Aβ_1–42_NF, indicating that its aggregates are well-distributed. Aβ_1–42_I had a size distribution peak at 384 nm, and Aβ_1–42_DM showed a wide particle size distribution comprising two peaks around 93 and 878 nm. These results suggested that increasing the steric hindrance of Tyr10 *via* molecular modification did not influence the aggregation of Aβ. Overall, Aβ_1–42_NT exhibited a smaller size peak and narrower size distribution than Aβ_1–42_ and its other mutants, consistent with the results obtained above.

## Discussion

Misfolding of Aβ into soluble and insoluble assemblies plays a critical role in the pathogenesis of AD. While, numerous evidence showed that amyloid plaques in the human AD contain a variety of post-translationally modified Aβ, including truncation, the E3- and E11-pyroglutarmation (Perez-Garmendia et al., 2010; Rijal Upadhaya et al., 2014), the D7-isomerization (Moro et al., 2018), the S8-phosphorylation (Kumar et al., 2013; Rijal Upadhaya et al., 2014), the S26-phosphorylation (Kumar et al., 2016), and the Y10-nitration of Aβ (Kummer et al., 2011). Most of them show a significant effect on the aggregation and increase neurotoxicity of Aβ *in vitro* and *in vivo*. Therefore, it is noticeable that detecting the effect of posttranslational modifications on the molecular properties of Aβ aggregates may contribute to understanding the relationship between Aβ misfolding and AD.

Interestingly, our previous studies showed that nitration of Aβ could inhibit its aggregation and reduce its neurotoxicity (Zhao et al., 2015, 2017, 2019). Moreover, tyrosine nitration ameliorated the aggregation and neurotoxicity of Aβ_1–42_ induced by Cu(II), and nitrated Aβ could protect neurons against the toxicity of Cu(II; Zhao et al., 2019). It is worth noting that nitrated Aβ is the only one that shows lower toxicity toward neurons compared with wild type Aβ. We also noticed that the levels of nitrate in the cerebrospinal fluid of AD patients notably decreased and how the decreased nitrite and nitrate levels affect the pathogenesis of AD remains unclear (Corzo et al., 2007). This evidence shows the importance of investigating the relationship between nitrated Aβ and AD. While the mechanism of nitration of Aβ inhibits its aggregation remains unknown.

This study found nitrotyrosine in the amyloid plaques in the cortex of 10-month-old female triple transgenic mice with AD (Figure 2). It indicated that Aβ might be nitrated *in vivo*. It has been reported that abnormally high levels of metal ions were found in amyloid plaques in the brain of AD patients (Faller and Hureau, 2009; Geng et al., 2012). It is already known that Aβ-Cu(II) and Aβ-heme complex are capable of catalyzing peroxynitrite production in the presence of hydrogen peroxide and nitrite (Lu et al., 2015; Zhao et al., 2015). Thus, nitrated Aβ can be found in the amyloid plaques (Giuffrida et al., 2009).

Conformational changes from α-helix to β-sheet are essential for the aggregation of Aβ (Qiu et al., 2015). As shown in Figure 3, after 48 h incubation, all the peptides showed a significant peak at near 1,240, which is the characteristic peak of β-sheet (Mikhonin et al., 2006). Moreover, the CD spectra also showed that there is little difference in the secondary structure at 48 h between the six peptides (Figure 4). It indicated that tyrosine modification has little impact on the conformational changes of Aβ. Tyrosine modification of Aβ affects its aggregation by altering the intermolecular interactions of Aβ.

Tyrosine nitration could increase the amino acid size of tyrosine and induce the deprotonation of the phenolic hydroxyl group of Tyr. As shown in Figure 1, the *pKa* value of the phenolic hydroxyl group of 2′,6′-dimethyltyrosine is 9.94, which is close to the *pKa* value of tyrosine (Table 1). It is hard for the phenolic hydroxyl group of 2′,6′-dimethyltyrosine to ionize at physiological conditions. The VW values of 2′,6′-dimethyltyrosine is bigger than that of tyrosine (Table 1), and the Aβ_1–42_DM and Aβ_1–42_I exhibited a higher tendency to aggregate compared with Aβ_1–42_. These results supported that increasing the amino acid size of tyrosine in Aβ would not inhibit its aggregation. By comparing the log D values of 2′,6′-dimethyltyrosine, iodotyrosine, and tyrosine (−0.47, −0.61, and −1.5, respectively), we found that 2′,6′-dimethyltyrosine and iodotyrosine are much more hydrophobic than tyrosine. According to the fibril structure of Aβ_1–42_ reported by Gremer et al. (2017), the hydrophobic clusters [(1) Ala2, Val36, Phe4, Leu34; (2) Leu17, Ile31, Phe19; and (3) Ala30, Ile32, Met35, Val40.] expand in the stacked subunits along the fibril axis and essentially contribute to fibril structure stability. Moreover, it has been reported that mutations at A2V (Ala is replaced with Val), and A2T (Ala is replaced with Thr) cause distinct changes in Aβ properties with A2V accelerating and A2T delaying its aggregation (Benilova et al., 2014). The cause of this phenomenon is attributed to the polarity of amino acids. Threonine is more polar than alanine and could destabilize the fibril by disrupting the hydrophobic cluster Ala2, Val36, Phe4, Leu34. On the contrary, valine is more hydrophobic than alanine and would strengthen the hydrophobic interaction leading to increased fibril stability (Gremer et al., 2017). Therefore, we propose that Aβ_1–42_DM and Aβ_1–42_I have a stronger propensity to aggregate because 2′,6′-dimethyltyrosine, and iodotyrosine are much more hydrophobic than tyrosine. It is well known that hydrogen bond interactions are important for maintaining the structure of the protein. We used Aβ_1–42_NF to test the effect of hydrogen bond interactions on the aggregation of Aβ. We found no significant difference between Aβ_1–42_ and Aβ_1–42_NF in ThT and Bis-ANS fluorescence intensity, the number of fibers, and aggregates size (Figures 5–8). Thus, it seems that nitration of Aβ inhibits its aggregation possibly by inducing the deprotonation of the phenolic hydroxyl group of Tyr10. Moreover, the percent deprotonation of nitrotyrosine, chlorotyrosine, and iodotyrosine is 84%, 23%, and 10%, respectively (Table 1). We also observed Aβ_1–42_I exhibited the highest ability to aggregation, followed by Aβ_1–42_Cl and Aβ_1–42_NT. These results further confirmed that the *pK_a_* changes of the phenolic hydroxyl group might be critical for the inhibitory effect (the *pK_a_* values of the phenolic hydroxyl group of iodotyrosine, chlorotyrosine, and nitrotyrosine are 8.34, 7.92, and 6.9, respectively; Table 1).

We also found that tyrosine chlorination could inhibit the aggregation of Aβ_1–42_. Chlorination of tyrosine could induce the deprotonation of the phenolic hydroxyl group of Tyr10 as well, and that’s how it could reduce the aggregation of Aβ_1–42_. Moreover, the Chlorination of Aβ can alter the secondary structure (Figures 3, 4). We speculated that chlorotyrosine may interact with the hydrophobic core of Aβ and alter its secondary structure. Chlorination of tyrosine is a commonly known consequence of myeloperoxidase activity at sites of inflammation (Nybo et al., 2019). Although it had not been reported that Aβ could be chlorinated *in vivo*. Oxidative stress is a key hallmark in AD, and many studies considered that oxidative stress is associated with generation (Butterfield and Boyd-Kimball, 2004; Aliev, 2011). Therefore, the relationship between tyrosine chlorination of Aβ and AD deserves more attention.

**Figure 9:**
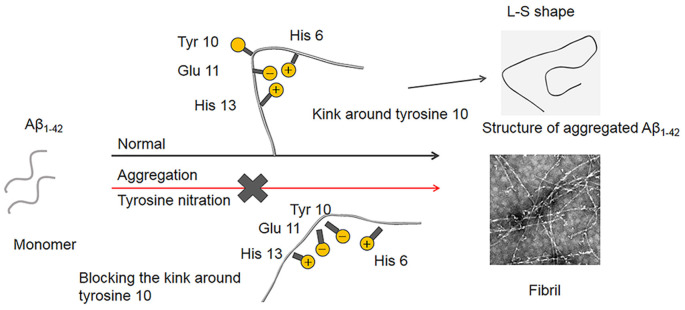
Proposed mechanism of Aβ nitration to inhibit its fibril formation. A single Aβ_1–42_ subunit forms an LS-shaped structure, in which the N-terminus is L-shaped, and the C-terminus is S-shaped. The kink around Tyr10 essentially contributes to the fibril stability of Aβ fibril. Nitration of Aβ could significantly decrease in the *pKa* of the phenol hydroxyl group in Tyr10, leading to the deprotonation of the phenolic hydroxyl group at physiological pH. As a result, the negatively charged Tyr10 could repel the negatively charged Glu11 and destroy the kink formed by the salt-bridge of Glu11 with His6 and His13, which prevents Aβ fibrilization and aggregation.

In terms of the structure of the Aβ_1–42_ fibrils, it has been demonstrated that a single Aβ_1–42_ subunit forms an LS-shaped structure, in which the N-terminus is L-shaped, and the C-terminus is S-shaped (Gremer et al., 2017). The Tyr10 residue is located at a kink in the N-terminal part of the β-sheet, and the salt-bridge between Glu11 with His6 and His13 stabilizes the kink. It is well known that mice do not naturally develop AD, and His13 is replaced by arginine in murine Aβ, which possibly prevents the formation of the kink, suggesting that the kink around Tyr10 is essential for the fibril formation of Aβ (Shivers et al., 1988; Fung et al., 2004; Lv et al., 2013). As noted above, the *pKa* decrease induced by Tyr10 nitration played a critical role in the inhibitory effect of nitration of Aβ on its aggregation. The phenolic hydroxyl group in nitrotyrosine is charged at physiological *pH*. Moreover, Guivernau et al. (2016) found that Aβ_E14_ (His14 is replaced with Glu, which is a negatively charged amino acid at physiological pH) exhibited a significantly lower propensity to aggregate compared with Aβ. Therefore, we proposed that nitration of Aβ could induce the deprotonation of the phenolic hydroxyl group in Tyr10 at physiological *pH*. The negatively charged hydroxyl group could repel its adjacent Glu11 to block the interaction between Glu11 and His6, thus destroy the kink around Tyr10. Consequently, Aβ fails to nucleate, and mature fibril formation is blocked (Figure 9). This study may also be used to explain our recent interesting findings that nitration of the tyrosine residues of human calcitonin will inhibit its aggregation (Ye et al., 2020).

## Conclusion

In conclusion, we systematically investigated the mechanism of nitration of Aβ to inhibit its aggregation. Our results showed that nitration of Aβ led to deprotonation of the phenolic hydroxyl group of Tyr10 at physiological *pH*, which played a decisive role in its inhibitory effect on Aβ aggregation. Nitration of Aβ at Tyr10 under physiological *pH* generated the negatively charged hydroxyl group that could repel the adjacent Glu11 and destroy the interaction among His6 or His13 and Glu11, leading to the disassembly of the kink around Tyr10. Consequently, Aβ failed to aggregate. These findings provide us new insights into the relation between Tyr10 nitration and Aβ aggregation, which would imply that keeping the balance of protein nitration *in vivo* is essential for preventing AD.

## Data Availability Statement

The original contributions presented in the study are included in the article, further inquiries can be directed to the corresponding author/s.

## Ethics Statement

The experiments and procedures were performed following the institutional guidelines regarding experimental animal use at Shenzhen University and were approved by the Animal Ethical and Welfare Committee of Shenzhen University (permit number AEWC-20140615-002).

## Author Contributions

QL and ZHG: conceptualization, resources, and supervision. JZ and QHS: data curation, formal analysis. QL: funding acquisition. JZ, QL, and ZHG: investigation, writing—review and editing. JZ, QHS, YZ, QLL, and ZJH: methodology. ZJ: project administration, writing—original draft. All authors have read and approved the final version submitted.

## Conflict of Interest

The authors declare that the research was conducted in the absence of any commercial or financial relationships that could be construed as a potential conflict of interest.

## Abbreviations

Aβ: amyloid-β
AD: Alzheimer’s disease
VW: van der waals volume
LogD: distribution coefficient
pKa: ionization constant
HFIP: hexa-fluoro-isopropanol
ThT: Thioflavin T
Bis-ANS: 4,4′-dianilino-1,1′-binaphthyl-5,5′-disulfonic acid
TEM: transmission electron microscopy
DLS: dynamic light scattering
AFM: atomic force microscopy
CD: circular dichroism
SERS: surface-enhanced Raman spectroscopy.
